# Robotic-assisted left carinal sleeve pneumonectomy with extracorporeal membrane oxygenation support for adenoid cystic carcinoma

**DOI:** 10.1016/j.xjtc.2025.07.025

**Published:** 2025-08-11

**Authors:** Camilo Moreno, Marina Paradela, Francisco Rivas, Carlos Déniz, Anna Muñoz, Ivan Macia, Ester Mendez, Raúl Herrera, David Toral, Amaya Ojanguren, Diego Gonzalez-Rivas

**Affiliations:** a Department of Thoracic Surgery, Bellvitge University Hospital, Hospitalet de Llobregat, Barcelona, Spain; b Department of Anesthesiology, Bellvitge University Hospital, Hospitalet de Llobregat, Barcelona, Spain; c Department of Cardiac Surgery, Bellvitge University Hospital, Hospitalet de Llobregat, Barcelona, Spain; d Minimally Invasive Thoracic Surgery Unit, Department of Thoracic Surgery, Coruña University Hospital, Coruña, Spain

**Figure:**
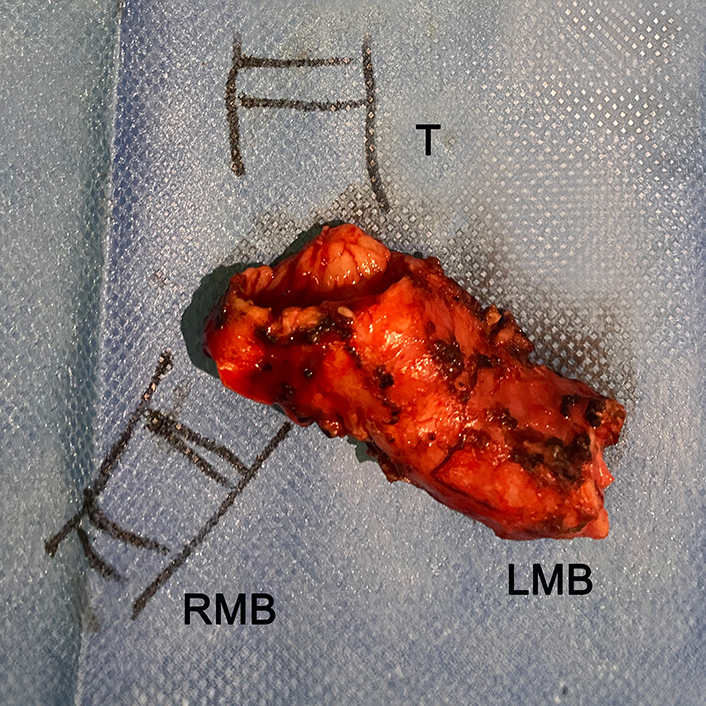
Macroscopic specimen showing the adenoid cystic carcinoma involving the carinal region.

**Central Message**

Left carinal sleeve pneumonectomy is one of the most complex procedures in thoracic surgery. A RATS approach with ECMO support is a feasible and safe minimally invasive alternative.

Sleeve pneumonectomy is one of the most challenging thoracic procedures as a result of airway reconstruction complexity. It is indicated for central tumors involving the main bronchus, carina, or distal trachea.^1^

This procedure has traditionally been performed via thoracotomy, sternotomy, or clamshell incision for access to the lower trachea and carina. Hybrid techniques combining thoracotomy and video-assisted thoracic surgery (VATS), as well as fully VATS-based approaches, have also been described.^2^^,^^3^ To date, no fully robotic approaches for this procedure have been documented. In this report, we describe the technical aspects of a left carinal sleeve pneumonectomy for adenoid cystic carcinoma of the lung using a robotic-assisted thoracic surgery (RATS) approach with extracorporeal membrane oxygenation (ECMO) support, highlighting its feasibility and safety.

## Surgical Technique

The patient was administered general anesthesia, and a right-sided double-lumen endotracheal tube was placed. Femorofemoral venous cannulation was performed for venovenous ECMO. A single 7500-unit dose of heparin was administered. ECMO support was initiated at the beginning and maintained throughout the procedure.

The patient was positioned in right lateral decubitus, with the da Vinci Xi system (Intuitive Surgical) in a left transverse thoracic setup. A 5-cm uniportal RATS incision was made in the fifth intercostal space along the anterior axillary line, anterior to the latissimus dorsi.

**Figure 1:**
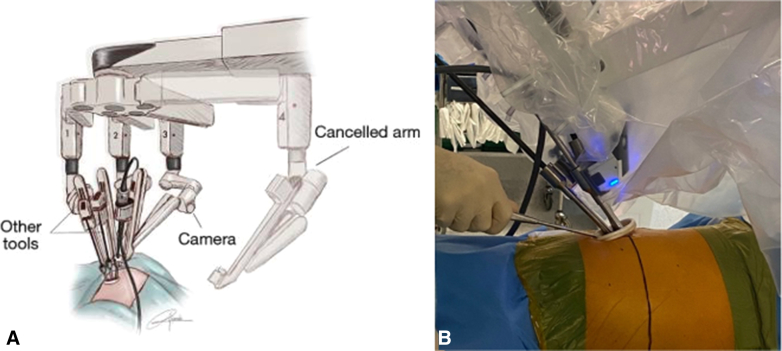
A, Robotic arm configuration on the left side. Reprinted from Gonzalez-Rivas and colleagues.^4^ B, Intraoperative view of instrument placement.

For the robotic setup, Arm 4 was deactivated. The camera was placed on Arm 3 (posterior angle), Arm 2 as the right hand (central), and Arm 1 as the left hand (anterior angle). This configuration follows the setup for uniportal RATS as described by Gonzalez-Rivas and colleagues^4^ (Figure 1, *A* and *B*).

### Left Pneumonectomy

**Figure 2:**
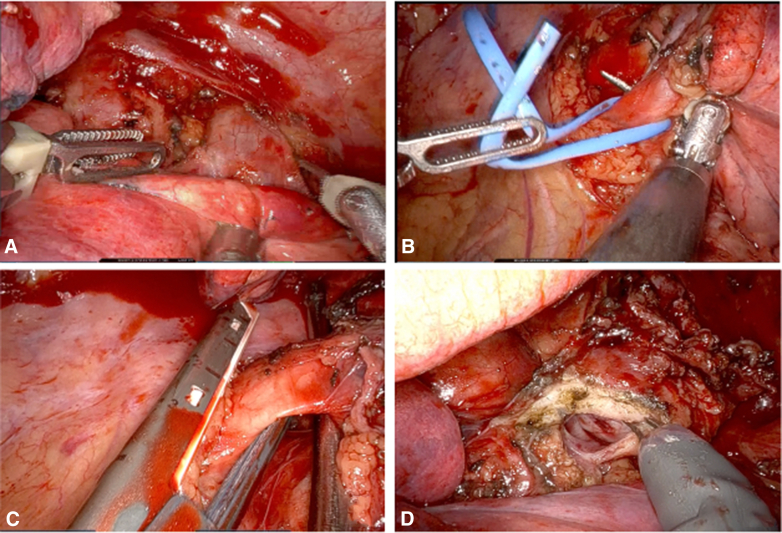
Division of (A) superior pulmonary vein (*SPV*), (B) inferior pulmonary vein (*IPV*), (C) left pulmonary artery (*LPA*), and (D) transection of the left main bronchus (*LMB*).

Dissection of the pulmonary hilum was performed, followed by separation of the esophagus with focal resection of the muscularis propria of the esophageal wall over an approximate length of 3 cm. The inferior and superior pulmonary veins (Figure 2, *A* and *B*) and the left pulmonary artery (Figure 2, *C*) were divided using an endoscopic vascular stapler. Subsequently, the distal transection of the left main bronchus was completed with robotic scissors (Figure 2, *D*), revealing tumor involvement up to the secondary lobar carina. The specimen was retrieved in an endobag without enlarging the incision.

### Carinal Dissection and Anastomosis

**Figure 3:**
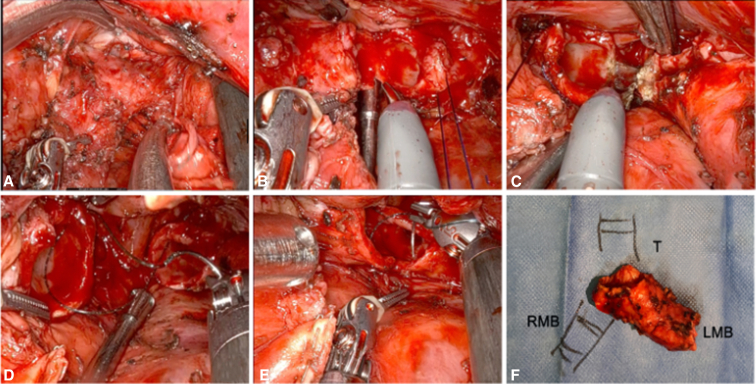
A, Exposure of the carina with traction using a suction instrument, displacing the esophagus and aorta. B, Section of the *RMB*. C, Section of the distal trachea. D-E, Primary end-to-end anastomosis. F, Resected specimen. *RMB*, Right main bronchus; *LMB*, left main bronchus.

The esophagus and aorta were posteriorly mobilized to expose the tracheal carina, assisted by the bedside surgeon using suction (Figure 3, *A*). Dissection extended from the distal trachea to right main bronchus (RMB), with safety sutures to prevent retraction.

The trachea and RMB were resected en bloc with the tumor (Figure 3, *B* and *C*). A residual lesion was identified in the RMB, requiring additional resection to the secondary carina to ensure macroscopically clear margins. Because this represented the maximal anatomically safe resection, no further margin was sent for frozen section.

A primary end-to-end anastomosis was performed between the trachea and the RMB using 2 continuous absorbable barbed 3-0 sutures (15-cm, 17-mm needle) (Figure 3, *D* and *E*). Anastomosis integrity was confirmed with a saline leak test. The resected specimen is shown in Figure 3, *F*.

Lymph nodes 5, 7, 10L, and 10R were excised. At the end of the procedure, the patient was extubated, and ECMO support was discontinued. The surgical procedure can be viewed in Video 1.

### Postoperative Outcomes

The postoperative course was uneventful: ambulation at 24 hours, drain removal at 48 hours, and discharge on day 5. Final pathology revealed an R1 resection attributable to an isolated microscopic infiltration in the RMB on frozen section, and the tumor was staged as pT4N0. After multidisciplinary discussion, adjuvant radiotherapy was indicated to complete treatment. At 6-month follow-up, the patient remains asymptomatic, with computed tomography scan showing no signs of recurrence.

## Discussion

This case suggests that combining RATS and ECMO support is a feasible and safe alternative for sleeve pneumonectomy. ECMO offers advantages by eliminating cross-field ventilation, improving surgical exposure and facilitating airway reconstruction without endotracheal interference.^5^

Left carinal sleeve pneumonectomy usually requires a bilateral approach, even with VATS techniques.^2^^,^^3^ RATS with ECMO allowed the procedure to be performed from a single side. Although a left-sided approach does not allow for right hilar release maneuvers, in this case the excellent exposure and 3-dimensional vision enabled wide dissection of the trachea and right main bronchus, minimizing anastomotic tension. Although promising, further studies are needed to evaluate reproducibility and compare outcomes with traditional techniques

### Webcast

You can watch a Webcast of this AATS meeting presentation by going to: https://www.aats.org/resources/robotic-left-carinal-sleeve-pn-9478.

**Figure:**
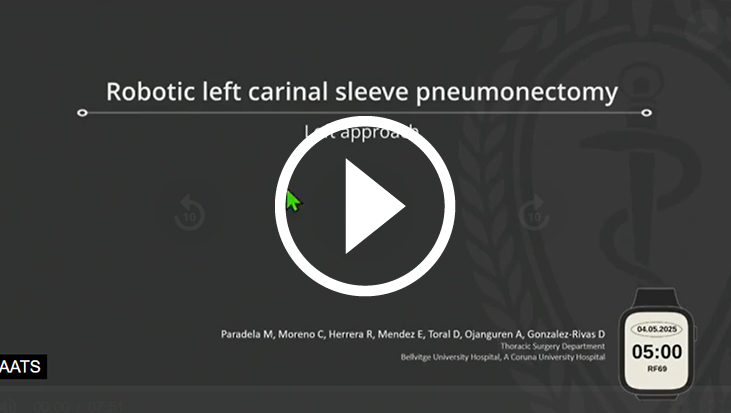

## Conflict of Interest Statement

The authors reported no conflicts of interest.

The *Journal* policy requires editors and reviewers to disclose conflicts of interest and to decline handling or reviewing manuscripts for which they may have a conflict of interest. The editors and reviewers of this article have no conflicts of interest.

## Supplementary Data

Video 1 Robotic-assisted left carinal sleeve pneumonectomy. Video available at: https://www.jtcvs.org/article/S2666-2507(25)00340-2/fulltext.
