# Supplementary figures and images for: Robotic-assisted left carinal sleeve pneumonectomy with extracorporeal membrane oxygenation support for adenoid cystic carcinoma
Source: JTCVS Tech. 2025 Aug 11;34:222–5. doi: 10.1016/j.xjtc.2025.07.025 (PMC12682967; doi:10.1016/j.xjtc.2025.07.025)

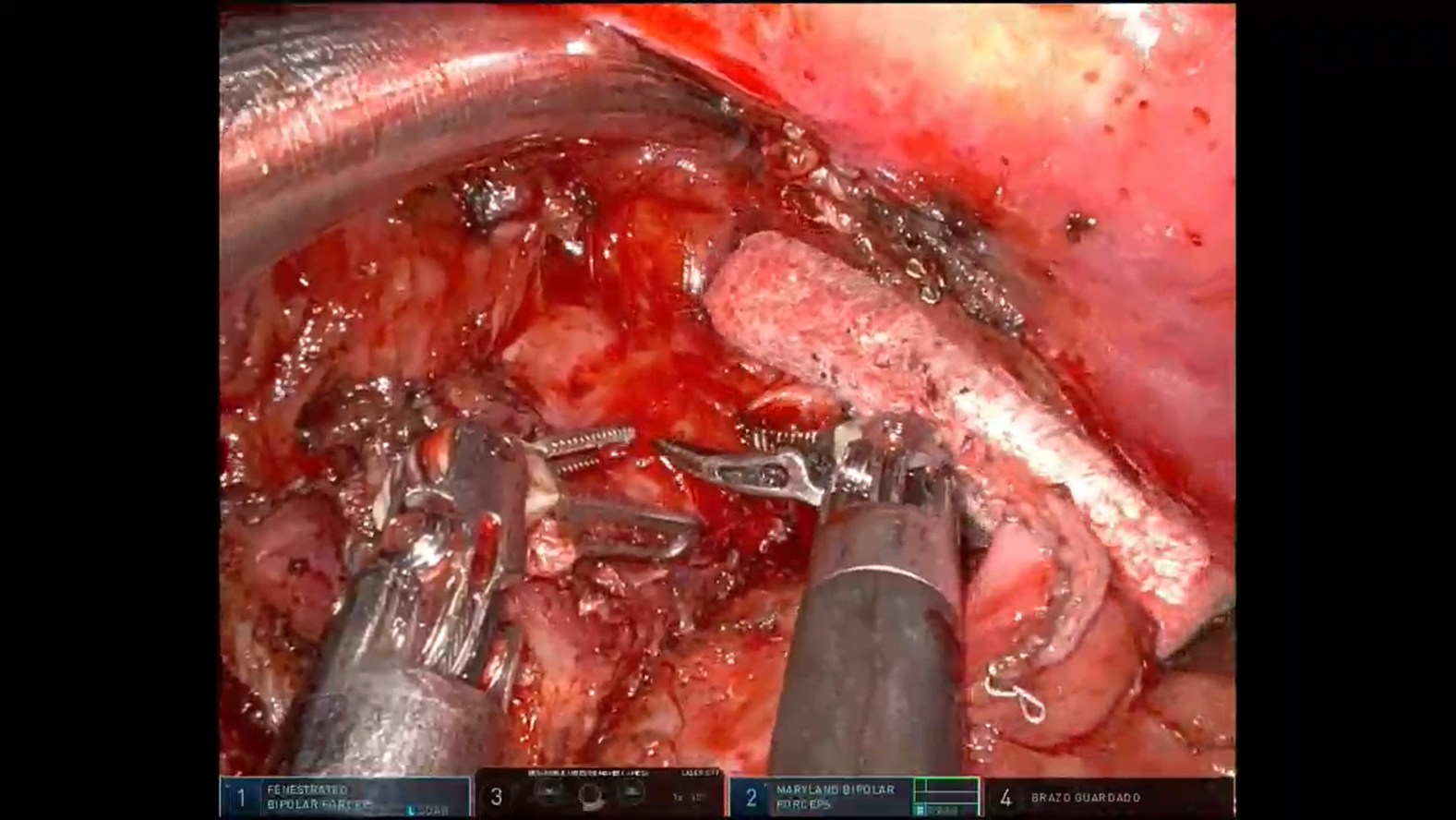

Supplement: Video 1 — Robotic-assisted left carinal sleeve pneumonectomy. Video available at: https://www.jtcvs.org/article/S2666-2507(25)00340-2/fulltext. [file fx3.jpg]
